# Association between aerobic capacity and the improvement in glycemic control after the exercise training in type 2 diabetes

**DOI:** 10.1186/s13098-017-0262-9

**Published:** 2017-08-18

**Authors:** Hideki Nojima, Masayasu Yoneda, Hiroshi Watanabe, Kiminori Yamane, Yoshihiro Kitahara, Kiyokazu Sekikawa, Hideya Yamamoto, Akihito Yokoyama, Noboru Hattori, Nobuoki Kohno, the Hiroshima University Health Promotion Study group, Akira Eboshida, Yasuo Hashimoto, Hiromi Kawasaki, Norihisa Kato, Hidemi Kurihara, Mitsuo Ochi, Megu Ohtaki, Kiyoshi Onari, Tsutomu Inamizu, Toshimasa Asahara, Ryozo Harada

**Affiliations:** 1 Department of Molecular and Internal Medicine, Institute of Biomedical & Health Sciences, Hiroshima University, 1-2-3 Kasumi, Minami-ku, Hiroshima, 734-8551 Japan; 2 Department of Sports Medicine, Hiroshima University, 1-2-3 Kasumi, Minami-ku, Hiroshima, 734-8551 Japan; 3 Department of Cardiovascular Medicine, Hiroshima University, 1-2-3 Kasumi, Minami-ku, Hiroshima, 734-8551 Japan; 4 Nojima Internal Medicine Clinic, 1-2-3 Kasumi, Minami-ku, Hiroshima, 734-8551 Japan; 5 Department of Endocrinology, Tsuchiya General Hospital, 3-30 Nakajima-cho, Naka-ku, Hiroshima 730-8655 Japan; 6 Chugoku Health Administration Center, Nippon Telegraph and Telephone West Corporation, 11-40 Hijiyama-honmachi, Minami-ku, Hiroshima, 732-0816 Japan; 7 Department of Respiratory Medicine, National Hospital Organization, Kure Medical Center, 3-1 Aoyama-cho, Kure, 736-0023 Japan; 8 Department of Hematology & Respiratory Medicine, Kochi University, Oko-cho Kohasu, Nankoku, Kochi 783-8505 Japan; 9 Hiroshima Cosmopolitan University, Ujina Campus, 5-13-18 Ujinanishi, Minami-ku, Hiroshima, Hiroshima 734-0014 Japan

**Keywords:** Aerobic exercise training, Accelerometer, Peak oxygen uptake, Type 2 diabetes

## Abstract

**Background:**

We investigated the influence of aerobic capacity on the improvement in glycemic control achieved by long-term aerobic exercise in type 2 diabetes.

**Methods:**

Fifty-three male patients with type 2 diabetes, recruited from outpatient clinics, wore multiple-memory accelerometers and were instructed to exercise at moderate intensity for ≥30 min on ≥3 days per week over 12 months. Peak oxygen uptake (peak ${\dot{\text{V}}\text{O}}_{ 2}$) and serum glycated albumin (GA) were measured at baseline and after 3, 6, 12 months. Peak ${\dot{\text{V}}\text{O}}_{ 2}$ data were expressed as percentages of predicted values.

**Results:**

According to the number of bouts of exercise (intensity, ≥4 METs; duration, ≥15 min), the subjects were divided into inactive (<3 times per week) or active (≥3 times per week) groups. Serum GA decreased significantly after 3, 6, 12 months only in the active group. When the subjects were assigned to four groups according to initial peak ${\dot{\text{V}}\text{O}}_{ 2}$ (%pred) (low-fitness or high-fitness) and the number of bouts of exercise (active or inactive), serum GA decreased significantly after 3, 6, 12 months only in the high-fitness/active group. When the subjects were also assigned to four groups according to the change in peak ${\dot{\text{V}}\text{O}}_{ 2}$ (%pred) (improved or unimproved) and the number of bouts of exercise (active or inactive), serum GA decreased significantly after 3 and 12 months only in the improved/active group.

**Conclusion:**

The improvement in glycemic control achieved by aerobic exercise was associated with both the initial and the increase in peak ${\dot{\text{V}}\text{O}}_{ 2}$ during aerobic exercise.

**Electronic supplementary material:**

The online version of this article (doi:10.1186/s13098-017-0262-9) contains supplementary material, which is available to authorized users.

## Background

Exercise plays a major role in the prevention [1] and control [2] of type 2 diabetes. Physical activity and aerobic capacity act as independent measures of exercise parameters. Lynch et al. reported that both moderately intense physical activity and high levels of aerobic capacity independently reduced the risk of type 2 diabetes in middle-aged men [3]. The Canadian physical activity longitudinal study also found an inverse association between aerobic capacity and the incidence of diabetes independent of the levels of leisure-time physical activity [4]. These two reports indicate that physical activity and aerobic capacity are independently associated with the risk of developing type 2 diabetes.

Peak oxygen uptake (peak ${\dot{\text{V}}\text{O}}_{ 2}$) is the gold standard measure of aerobic capacity [5]. Peak ${\dot{\text{V}}\text{O}}_{ 2}$ is reduced in type 2 diabetic patients compared with non-diabetic subjects [6], and a low peak ${\dot{\text{V}}\text{O}}_{ 2}$ strongly predicts long-term cardiac mortality in type 2 diabetic patients [7]. Seibaek et al. also reported an inverse correlation between insulin resistance and peak ${\dot{\text{V}}\text{O}}_{ 2}$ in type 2 diabetic patients [8]. Furthermore, aerobic exercise significantly increases peak ${\dot{\text{V}}\text{O}}_{ 2}$ [9] and improves glycemic control in type 2 diabetic patients [10, 11]. Larose et al. reported that improvements in maximal aerobic fitness were significantly related with improvements in HbA1c with aerobic training only and with combined aerobic and resistance training [12]. However, the influence of aerobic capacity on the improvement in glycemic control associated with aerobic exercise training in type 2 diabetic patients has yet to be conclusively established.

The Hiroshima University Health Promotion Study was an exercise training study, the main purpose of which was to assess the cardiovascular, metabolic, and hormonal responses to aerobic exercise training in type 2 diabetic patients. We previously reported that moderate-intensity aerobic exercise training over 12 months reduced oxidative stress and improved glycemic control in type 2 diabetic patients [13]. In the present study, we instructed patients with type 2 diabetes to perform aerobic exercise training over a 12-month period, and peak ${\dot{\text{V}}\text{O}}_{ 2}$ and serum glycated albumin (GA) levels were measured at baseline and after 3, 6, 12 months to determine the effect of aerobic capacity on glycemic control.

## Methods

### Subjects

The study participants consisted of 62 male patients with type 2 diabetes (age range, 35–74 years) recruited from outpatient clinics between January and July, 2003, 55 of whom were considered eligible. We collected follow-up data from January, 2003 until August, 2004. Diabetes was defined according to established criteria [14]. The exclusion criteria included (1) hemoglobin A1C ≥10% (82.94 mmol/mol), (2) clinical findings of diabetic micro- or macro-vascular complications, (3) taking insulin therapy, (4) inability to walk for exercise, (5) medical conditions potentially contraindicating the exercise program, and (6) obstructive or restrictive abnormalities in spirometry: [forced expiratory volume in one second (FEV_1_)/forced vital capacity (FVC)] <70% or {percent predicted value of FVC [FVC (%pred)]} <80%. Two participants withdraw from the study due to low back pain limiting exercise training (1 patient) and respiratory tract infection (1 patient). The remaining 53 patients were included in analysis.

### Ethics approval and consent to participate

The study protocol, which is in accordance with the Declaration of Helsinki, was approved by the Ethics Committee of Hiroshima University and written informed consent was obtained from all participants prior to commencement of the study.

### Clinical examination

Information on the duration of diabetes and smoking status was collected at baseline. Anthropometry (height and body weight), blood pressure measurement, blood sampling and cardiopulmonary exercise testing were performed at baseline and after 3, 6, 12 months of the aerobic training program. Blood pressure were measured using an automatic pulse-wave velocimeter (Form PWV/ABI, model BP-203RPE, Japan Colin Cooperation). Body mass index was calculated by dividing weight (in kilograms) by height (in meters) squared. Total body fat (%) was assessed by bioimpedance measurements (TBF-501; Tanita, Tokyo, Japan). At baseline and after 12 months, a registered dietitian calculated caloric intake using food frequency questionnaire software, Excel Eiyoukun FFQg (v 1.0) (Kenpousha Co. Ltd., Tokyo, Japan). Venous blood samples were taken after meals prior to an exercise test to prevent hypoglycemia during the exercise test. Total cholesterol, high-density lipoprotein (HDL) cholesterol, triglyceride, and HbA1c were measured at the respective outpatient clinics. Serum glycated albumin (GA) was measured by high performance liquid chromatography [15]. C-reactive protein (CRP) was measured using latex-enhanced immunonephelometric assays [16] on a BNII analyzer (Dade Behring, Tokyo, Japan).

Serum GA was measured to monitor glycemic control. Serum GA levels reflect overall glycemic control during the previous 2 weeks, whereas HbA1c provides an integrated measurement of blood glucose during the previous 2–3 months [17, 18]. Schleicher et al. have proposed that glycated serum protein is a more sensitive index than HbA1c, possibly as a consequence of the higher albumin content in serum [19]. Ueda et al. also found that there was greater glucose binding over time in GA than HbA1c for all glucose concentrations [20]. Yoshiyuki et al. demonstrated that GA is a better indicator for glucose excursion than HbA1c in type 2 diabetes [21]. Therefore, serum GA was used as a sensitive marker of glycemic control because the subjects in this study did not have markedly abnormal glycemic control.

### Spirometry

FEV_1_ and FVC were measured at baseline and after 12 months by an experienced technician using HI-701 (Chest Co., Tokyo, Japan) or SUPER SPIRO DISCOM-21 FXП (Chest Co., Tokyo, Japan), respectively. Spirometric maneuvers were performed according to recommendations of the American Thoracic Society [22]. FEV_1_ data were reported in the absolute values and represented as FEV_1_ (%pred) as calculated by Berglund’s equation [23]. FVC data were reported in absolute values and expressed as FVC (%pred) as calculated by Baldwin’s equation [24].

### Determination of peak oxygen uptake

All participants underwent a cardiopulmonary exercise test using a bicycle ergometer (Ergometer STB-2400: Nihon Kohden Co., Tokyo, Japan). After a sufficient period of rest on the ergometer, exercise was started with a 1-min warm-up at 10 W, followed by the ramp protocol (20 W/min). An electrocardiogram and heart rates were recorded during the test using an electrocardiograph (QP932D: Nihon Kohden Co., Tokyo, Japan). ${\dot{\text{V}}\text{O}}_{ 2}$ was measured using a respiratory gas-exchange analyzer (AE300SRC: Minato Medical Science Co. Ltd., Osaka, Japan). The exercise test was terminated when any of the following conditions were observed: (1) the subject’s predicted maximum heart rate [220—age (years)] was achieved, (2) detection of ischemic signs in the electrocardiogram, or (3) the subject could no longer sustain a pedaling cadence of at least 50 revolutions per minute due to dyspnea, leg fatigue, or other symptoms [25]. Peak ${\dot{\text{V}}\text{O}}_{ 2}$ was estimated by extrapolating to the estimated maximal heart rate when heart rate was plotted against ${\dot{\text{V}}\text{O}}_{ 2}$. Peak ${\dot{\text{V}}\text{O}}_{ 2}$ data was reported in absolute values and expressed as a percentage of the predicted value [peak ${\dot{\text{V}}\text{O}}_{ 2}$ (%pred)]. In this study, the peak ${\dot{\text{V}}\text{O}}_{ 2}$ (%pred) was calculated using the formula for Japanese male equation as follows: Peak ${\dot{\text{V}}\text{O}}_{ 2}$ = 51.445−0.331 × age (years) [26]. We used the percentage of predicted value because we assess the effect of initial peak ${\dot{\text{V}}\text{O}}_{ 2}$ on improvements in glycemic control achieved by aerobic exercise training.

### Exercise training protocol and assessment of physical activity

All participants were requested to perform aerobic exercise for ≥30 min on ≥3 days per week at baseline, with follow-up requests after 3, 6, and 12 months. The American College of Sports Medicine and the American Diabetes Association recommend moderate-to-vigorous intensity aerobic exercise in type 2 diabetic patients [27], accordingly our study’s exercise intensity was targeted at 50% of peak ${\dot{\text{V}}\text{O}}_{ 2}$ using a pulse rate monitor (6102, Tanita, Tokyo, Japan). The recommended types of aerobic exercise were walking and jogging. No specific advice was given to the subjects concerning dietary habits during the study period. All subjects received regular treatment for diabetes mellitus at their outpatient clinics.

To examine the subjects’ physical activity, pedometers with multiple-memory uniaxial accelerometers (Life Corder; Suzuken Co. Ltd, Nagoya, Japan) were used. This device records the number of footsteps and 10-level exercise intensity every 4 s, based on the amplitude and frequency of accelerations in the vertical direction. The exercise intensity calculated by the device corresponds with daily activities as estimated by a time-motion study, and it also correlates with overall energy expenditure as determined by whole-body indirect calorimetry or breath gas analysis [28]. All participants were requested to wear the accelerometer over the right or left hip throughout the day, to follow their usual routine of daily activities and to remove the pedometer only when bathing, showering or sleeping. The data of the accelerometer were retrieved at baseline and after 3, 6, 12 months of the aerobic training program. We used physical activity data every day for statistical analysis.

The duration, frequency, and intensity of physical activity were classified using the pedometers described above. The study subjects were divided into inactive (<3 times per week, n = 30) and active (≥3 times per week, n = 23) groups according to the number of exercise bouts (intensity, ≥4 METs; duration, ≥15 min), as 50% of initial peak ${\dot{\text{V}}\text{O}}_{ 2}$ was equivalent to 3.7 ± 0.7 METs in our study. To assess the effect of initial peak ${\dot{\text{V}}\text{O}}_{ 2}$ on improvements in glycemic control achieved by aerobic exercise training, the subjects were also assigned to groups according to peak ${\dot{\text{V}}\text{O}}_{ 2}$ (%pred) measured at baseline, including low-fitness [<median peak ${\dot{\text{V}}\text{O}}_{ 2}$ (%pred) of all subjects: 100.2%] or high-fitness [>median peak ${\dot{\text{V}}\text{O}}_{ 2}$ (%pred) of all subjects] groups. Furthermore, to determine the effect of the increase in peak oxygen uptake on glycemic control, the study subjects were also divided into unimproved [reduced peak ${\dot{\text{V}}\text{O}}_{ 2}$ (%pred) after 12 months] and improved [increased peak ${\dot{\text{V}}\text{O}}_{ 2}$ (%pred) after 12 months] groups.

### Statistical analysis

Differences in categorical variables between the groups before intervention were analyzed using the Chi square test, while continuous variables were tested by analysis of covariance (ANCOVA) adjusted for age, followed by a Bonferroni multiple comparison test. Serum triglyceride level was log-transformed because of a skewed data distribution. Repeated measures ANOVA models were used to analyze, followed by a Bonferroni multiple comparison test. Repeated measures two-way (time × group) ANOVA models were used to analyze the effect of intervention on outcome measures to assess the difference between the groups. *P* values of <0.05 were considered to be statistically significant. The statistical tests were performed using the SPSS 12.0 J software program (SPSS Japan, Inc., Tokyo, Japan).

## Results

The subjects in the active group were significantly older than those in the inactive group, while serum HDL cholesterol level at baseline was significantly higher in the active group than in the inactive group after adjusting for age (Table 1). However, the other baseline characteristics were similar between the two study groups.

**Table 1:** Characteristics of the study participants at baseline and exercise volume of the study participants during the study period

Characteristics	Inactive group (n = 30)	Active group (n = 23)	*P* value
Age (years)	51.9 ± 10.1	57.6 ± 7.8	0.031
Duration of diabetes (years)	6.1 ± 4.5	7.7 ± 6.3	0.298
Smoking status (never/past/current)	6/14/10	6/11/6	0.081
Diabetes treatment			
Sulphonylureas	10	9	0.575
α glucosidase inhibitors	9	7	0.888
Biguanides	6	1	0.107
Thiazolidines	2	0	0.217
Glinides	2	0	0.217
Body mass index (kg/m^2^)	25.9 ± 4.4	23.7 ± 3.5	0.185
Total body fat (%)	25.5 ± 7.6	21.9 ± 6.2	0.342
Lean body mass (kg)	52.7 ± 6.0	50.0 ± 6.4	0.250
Systolic blood pressure (mmHg)	139.2 ± 16.9	140.2 ± 21.2	0.942
Diastolic blood pressure (mmHg)	85.6 ± 9.5	84.1 ± 11.3	0.707
Total cholesterol (mmol/l)	5.31 ± 0.85	5.32 ± 0.97	0.509
Triglyceride^a^ (mmol/l)	1.51 (1.04–2.32)	1.46 (0.88–1.90)	0.958
HDL cholesterol (mmol/l)	1.23 ± 0.25	1.46 ± 0.33	0.022
HbA1c (%)	7.5 ± 1.3	7.5 ± 1.0	0.700
Glycated albumin (%)	21.7 ± 4.8	23.5 ± 4.0	0.256
Peak ${\dot{\text{V}}\text{O}}_{ 2}$ (ml/min/kg)	25.6 ± 5.2	27.0 ± 4.1	0.057
Peak ${\dot{\text{V}}\text{O}}_{ 2}$ (%pred) (%)	97.3 ± 18.9	106.9 ± 15.1	0.052
FVC (%pred) (%)	110.5 ± 13.4	106.0 ± 12.8	0.366
FEV_1_ (%pred) (%)	105.3 ± 13.2	107.8 ± 14.3	0.766
FEV_1_/FVC (%)	78.8 ± 4.9	78.9 ± 4.2	0.369
Number of steps (/day)	6477 ± 2586	11,690 ± 4842	<0.001
Number of exercise bouts (/week)	0.9 ± 0.8	8.8 ± 6.6	<0.001

Results were expressed as mean ± SD or median (interquartile range)

Values were analyzed by analysis of covariance (ANCOVA) with age as the covariate

^a^Analyses performed on the natural logarithm

**Fig. 1:**
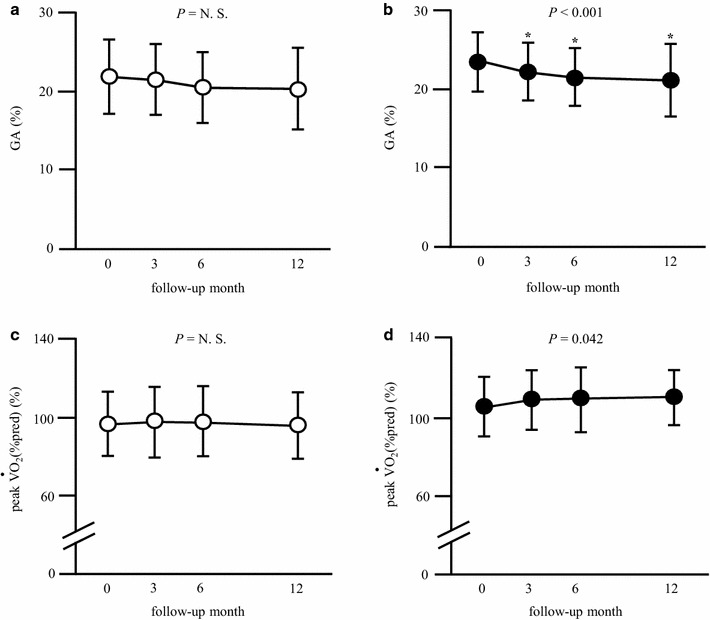
Effect of aerobic exercise on glycemic control and peak ${\dot{\text{V}}\text{O}}_{ 2}$. **a**, **c** The inactive groups; **b**, **d** the inactive groups. The results are expressed as mean ± SD. *P < 0.05 vs. baseline. Inactive group (n = 30), active group (n = 23) *GA* glycated albumin, *N.S.* not significant

Figure 1 shows that serum GA levels decreased significantly after 3, 6, 12 months in the active group, whereas no significant change was observed in the inactive group. Peak ${\dot{\text{V}}\text{O}}_{ 2}$ (%pred) increased after 12 months in the active group, but remained unchanged in the inactive group. There was no significant change in the caloric intake during the study period in any group (Additional file 1: Table S1).

**Fig. 2:**
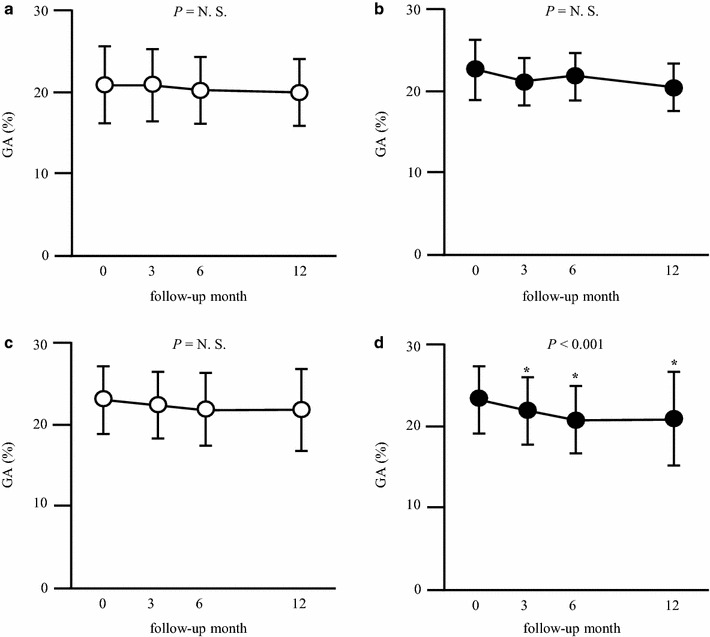
Effect of initial peak ${\dot{\text{V}}\text{O}}_{ 2}$ on change in glycemic control resulting from aerobic exercise. **a** Low-fitness/inactive group (n = 19), **b** low-fitness/active group (n = 7), **c** high-fitness/inactive group (n = 11), **d** high-fitness/active group (n = 16). The results are expressed as mean ± SD. *P < 0.05 vs. baseline. *GA* glycated albumin, *N.S*. not significant

**Fig. 3:**
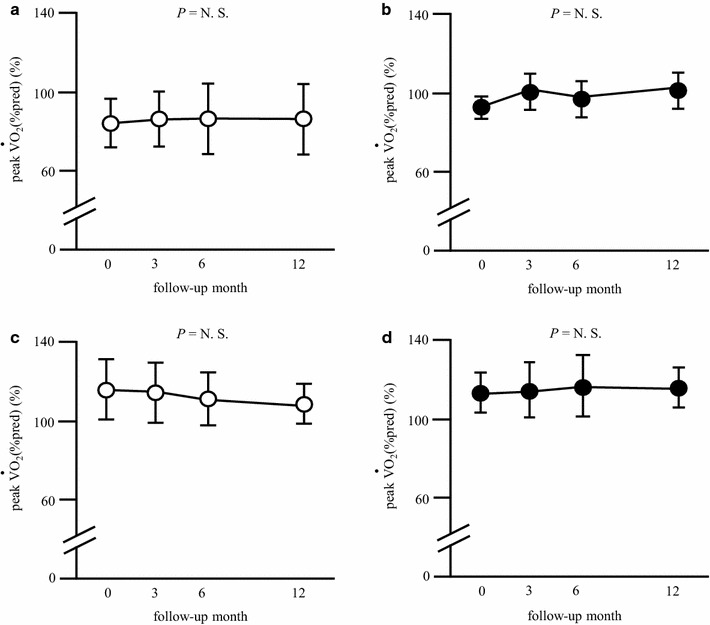
Effect of initial peak ${\dot{\text{V}}\text{O}}_{ 2}$ on change in peak ${\dot{\text{V}}\text{O}}_{ 2}$ resulting from aerobic exercise. **a** Low-fitness/inactive group (n = 19), **b** low-fitness/active group (n = 7), **c** high-fitness/inactive group (n = 11), **d** high-fitness/active group (n = 16). The results are expressed as mean ± SD. *P < 0.05 vs. baseline. *N.S.* not significant

Subsequently, the subjects were subdivided into low-fitness/inactive (n = 19), low-fitness/active (n = 7), high-fitness/inactive (n = 11), and high-fitness/active groups (n = 16). The baseline characteristics of the study subjects are shown in Additional file 1: Table S2. Although the systolic blood pressure was significantly higher in the low-fitness/active group compared with the high-fitness/active group (*P* = 0.035), the other baseline characteristics did not differ among the four study groups. Serum GA levels significantly decreased after 3, 6, 12 months only in the high-fitness/active group, whereas they remained unchanged in the other three groups (Fig. 2). Analysis by repeated measures two-way ANOVA model showed that there was no intergroup difference in the change in GA levels (P = 0.599). Peak ${\dot{\text{V}}\text{O}}_{ 2}$ (%pred) showed a trend to increase in the low-fitness/active group (*P* = 0.085) but did not change in the other three groups (Fig. 3). Serum CRP levels significantly increased after 3, 6, 12 months only in the high-fitness/inactive group, whereas they remained unchanged in the other three groups (Additional file 1: Table S3).

**Fig. 4:**
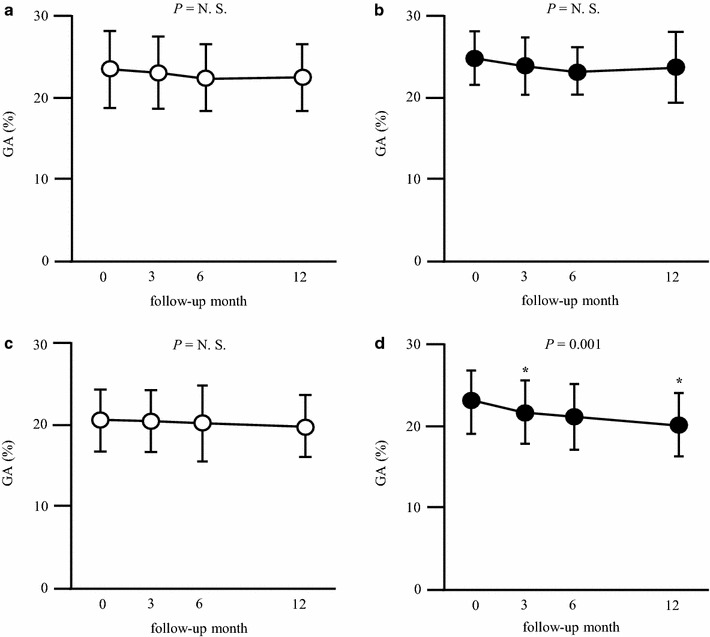
Effect of increase in peak ${\dot{\text{V}}\text{O}}_{ 2}$ on change in glycemic control resulting from aerobic exercise. **a** Unimproved/inactive group (n = 13), **b** unimproved/active group (n = 8), **c** improved/inactive group (n = 17), **d** improved/active group (n = 15). The results are expressed as mean ± SD. *P < 0.05 vs. baseline. *GA* glycated albumin, *N.S.* not significant

Furthermore, the subjects were subdivided into unimproved/inactive (n = 13), unimproved/active (n = 8), improved/inactive (n = 17), and improved/active groups (n = 15). Baseline HDL cholesterol level was significantly higher in the improved/active compared with the improved/inactive group (*P* = 0.039), while the other baseline characteristics did not differ among the four groups (Additional file 1: Table S4). Serum GA levels decreased significantly after 3 and 12 months only in the improved/active group, whereas they did not change in the other three groups (Fig. 4). Analysis by repeated measures two-way ANOVA model showed that there was no intergroup difference in the change in GA levels (P = 0.669).

## Discussion

The present study is quite unique in that the initial peak ${\dot{\text{V}}\text{O}}_{ 2}$ was a major factor for the improvement in glycemic control achieved by ≥3 aerobic exercise bouts per week in male type 2 diabetic patients. Furthermore, the increase in peak ${\dot{\text{V}}\text{O}}_{ 2}$ brought about by long-term aerobic exercise training was associated with the improvement in glycemic control only in the improved/active group.

After 3, 6, 12 months of exercise training, a significant improvement in glycemic control was observed only in subjects categorized as the high-fitness/active group. This suggests that high baseline peak ${\dot{\text{V}}\text{O}}_{ 2}$, in addition to aerobic exercise training, is important for improving glycemic control. Previous studies have shown a close relationship between aerobic capacity and incidence of type 2 diabetes, independent of physical activity levels [3, 4]. In contrast, the present study demonstrated the degree of aerobic capacity affected the changes in glycemic control associated with aerobic exercise training in type 2 diabetic patients. Oxygen is taken into the lung by respiration and transported to working muscles to be utilized during aerobic exercise [29]. A sufficient oxygen uptake is required to aerobically convert glucose into energy during aerobic exercise. The fact may explain the association between initial peak ${\dot{\text{V}}\text{O}}_{ 2}$ and the improvement of glycemic control achieved by aerobic exercise training, but the mechanism could not be fully elucidated.

The present study did not show any change in glycemic control in the subjects categorized to the low-fitness/active group. The effect of accumulated short exercise bouts, consisting of more than three bouts of ≥15 min duration of intermediate- or high-intensity aerobic exercise each week, was evaluated. The numbers of steps per day and number of exercise bouts (intensity, ≥4 METs; duration, ≥15 min) per week were similar between the low-fitness/active group and the high-fitness/active group (Table 1). The low-fitness male type 2 diabetic patients might thus require more bouts, a longer duration or higher intensity of aerobic exercise to trigger the cascade of mitochondrial genesis and improve glycemic control than that used in this study.

Furthermore, this study found a significant improvement in glycemic control after 12 months of exercise training only in subjects assigned to the improved/active group. This indicates that an increase in peak ${\dot{\text{V}}\text{O}}_{ 2}$ following aerobic exercise training is an important factor contributing to the improvement of glycemic control in type 2 diabetic patients. McMurray et al. reported that increased aerobic capacity after a 9-week course of aerobic exercise training was associated with a reduction in cardiovascular risk factors, including hypercholesterolemia and hypertension [30]. The current study demonstrated that increased peak ${\dot{\text{V}}\text{O}}_{ 2}$ is also important for improving hyperglycemia, one of the cardiovascular risk factors.

Several potential mechanisms may be involved in the beneficial effect of increased peak ${\dot{\text{V}}\text{O}}_{ 2}$ on glycemic control. Previous studies have shown that skeletal muscle characteristics such as decreased proportion of type I muscle fibers and capillary density [31], increased muscle lipid content [32], and a higher glycolytic to oxidative enzyme ratio [33] may be associated with decreased insulin sensitivity. These abnormalities potentially contribute to the lower peak ${\dot{\text{V}}\text{O}}_{ 2}$ values often seen in type 2 diabetic patients. Moreover, there is evidence that aerobic exercise training increases skeletal muscle capillary density, thereby improving oxidative capacity in these muscles [34]. This suggests that aerobic exercise training increases oxygen uptake and enhances oxygen utilization at the level of skeletal muscle, thus increasing skeletal muscle oxidative capacity and, ultimately, increasing insulin sensitivity and improving glycemic control.

There are some limitations of the present study. First, sample size was relatively small, especially the low fitness/active group. Therefore significant improvement in GA could not have been observed in the low fitness/active group. Second, exercise training in this study was not performed under the supervision of a trained investigator, and this study may be considered as assessing the effects of exercise in a naturalistic environment. In this study, the subjects’ actual physical activity was quantified over a 12-month period using pedometers with multiple-memory uniaxial accelerometers, and we investigated the long-term efficacy of short bouts of aerobic exercise for ≥15 min per bout at least 3 times a week over a 12-month period in type 2 diabetic patients.

## Conclusions

The initial peak ${\dot{\text{V}}\text{O}}_{ 2}$ and the increase in peak ${\dot{\text{V}}\text{O}}_{ 2}$ achieved by our study’s aerobic exercise training protocol could be an important role in the improvement of glycemic control in male type 2 diabetic patients. Furthermore, short bouts of exercise could enhance aerobic capacity and improve glycemic control in type 2 diabetes with long-term aerobic exercise training. Further studies are needed to elucidate the association between aerobic capacity and the improvement in glycemic control after the exercise training in type 2 diabetes because analysis by repeated measures two-way ANOVA model showed that there was no intergroup difference in the change in GA levels in our study.

## Additional file

**Additional file 1: Table S1.** Caloric intake of the study participants during the study period. **Table S2.** Characteristics of the study subjects at baseline and exercise volume of the study participants during the study period. **Table S3.** CRP of the study participants during the study period. **Table S4.** Characteristics of the study subjects at baseline and exercise volume of the study participants during the study period.

## Abbreviations

CRP: C-reactive protein
FEV_1_: forced expiratory volume in one second
FVC: forced vital capacity
HDL cholesterol: high-density lipoprotein cholesterol
GA: glycated albumin
peak ${\dot{\text{V}}\text{O}}_{ 2}$: peak oxygen uptake
