# Supplementary material for: Association between aerobic capacity and the improvement in glycemic control after the exercise training in type 2 diabetes
Source: Diabetol Metab Syndr. 2017 Aug 18;9:63. doi: 10.1186/s13098-017-0262-9 (PMC5563031; doi:10.1186/s13098-017-0262-9)
Supplement: Supplementary file 1 — Additional file 1: Table S1. Caloric intake of the study participants during the study period. Table S2. Characteristics of the study subjects at baseline and exercise volume of the study participants during the study period. Table S3. CRP of the study participants during the study period. Table S4. Characteristics of the study subjects at baseline and exercise volume of the study participants during the study period. [file 13098_2017_262_MOESM1_ESM.docx]

**Additional files**

| **Table S1. Caloric intake of the study participants during the study period.** | | | |
| --- | --- | --- | --- |
| **Characteristics** | **at baseline** | **aftter 12 months** | ***P* value** |
| Inactive Group (n = 30) | 1811.3± 291.7 | 1799.7 ± 339.3 | 0·820 |
| Active Group (n = 23) | 1728.2 ± 451.3 | 1707.1± 359.9 | 0.736 |

Note: Results were expressed as mean ± SD.

Values were analyzed by repeated measures ANOVA models were used to analyze.

| **Table S2. Characteristics of the study subjects at baseline and exercise volume of the study participants**  **during the study period.** | | | | | | |
| --- | --- | --- | --- | --- | --- | --- |
| **Characteristics** | **low-fitness/inactive**  **(n = 19)** | **low-fitness/active**  **(n = 7)** | **high-fitness/inactive**  **(n = 11)** | **high-fitness/active**  **(n = 16)** | ***P* value** |  |
| Age (years) | 53.2 ± 10.3 | 57.4 ± 7.8 | 49.7 ± 9.7 | 57.6 ± 8.1 | 0.138 |  |
| Duration of diabetes (years) | 6.0 ± 4.2 | 7.9 ± 7.2 | 6.5 ± 5.3 | 7.7 ± 6.2 | 0.945 |  |
| Smoking status (never/past/current) | 4/9/6 | 3/2/2 | 2/5/4 | 3/9/4 | 0.858 |  |
| Diabetes treatment |  |  |  |  |  |  |
| Sulphonylureas | 8 | 2 | 2 | 7 | 0.439 |  |
| α glucosidase inhibitors | 7 | 2 | 2 | 5 | 0.751 |  |
| biguanides | 2 | 0 | 4 | 1 | 0.079 |  |
| thiazolidines | 2 | 0 | 0 | 0 | 0.306 |  |
| glinides | 2 | 0 | 0 | 0 | 0.306 |  |
| Body mass index (kg/m^2^) | 26.4 ± 4.7 | 25.5 ± 4.3 | 25.2 ± 4.0 | 22.9 ± 3.0 | 0.180 |  |
| Total body fat (%) | 26.0 ± 8.7 | 24.4 ± 6.7 | 24.6 ± 5.6 | 20.9 ± 5.9 | 0.361 |  |
| Lean body mass (kg) | 53.1 ± 6.3 | 50.6 ± 6.0 | 52.1 ± 5.4 | 49.8 ± 6.8 | 0.627 |  |
| Systolic blood pressure (mmHg) | 141.7 ± 18.0 | 157.7 ± 17.6 | 134.8 ± 14.7 | 133.6 ± 18.9^†^ | 0.037 |  |
| Diastolic blood pressure (mmHg) | 86.4 ± 11.0 | 92.0 ± 6.6 | 84.3 ± 6.5 | 81.1 ± 11.4 | 0.148 |  |
| Total cholesterol (mmol/l) | 5.21 ± 0.89 | 4.74 ± 1.14 | 5.49 ± 0.80 | 5.59 ± 0.77 | 0.509 |  |
| Triglyceride^†^ (mmol/l) | 1.51 (1.11–2.22) | 1.42 (0.90–2.13) | 1.58 (0.94–2.67) | 1.46 (0.85–1.88) | 0.151 |  |
| HDL cholesterol (mmol/l) | 1.27 ± 0.24 | 1.35 ± 0.26 | 1.15 ± 0.26 | 1.51 ± 0.36 | 0.060 |  |
| A1C (%) | 7.0 ± 1.2 | 7.2 ± 0.8 | 7.3 ± 1.4 | 7.1 ± 1.1 | 0.947 |  |
| Glycated albumin (%) | 20.7 ± 4.6 | 22.9 ± 3.8 | 23.4 ± 5.0 | 23.7 ± 4.1 | 0.244 |  |
| CRP^†^ (mg/l) | 0.10(0.04-0.23) | 0.03(0.02-0.16) | 0.03(0.02-0.13) | 0.04(0.02-0.08)) | 0.062 |  |
| Peak O_2_ (ml/min/kg) | 22.6 ± 3.0 | 22.9± 1.9 | 30.9± 3.9* ** | 28.8± 3.4* ** | <0.001 |  |
| Peak O_2_ (%pred) (%) | 86.5 ± 9.4 | 90.5± 4.8 | 116.1± 16.4* ** | 114.1 ± 12.0* ** | <0.001 |  |
| FVC (%pred) (%) | 108.4 ± 11.3 | 104.8± 13.2 | 114.0± 16.4 | 109.1 ± 12.8 | 0.423 |  |
| FEV_1_ (%pred) (%) | 103.2 ± 10.5 | 107.0± 16.6 | 109.1 ± 16.9 | 108.1 ± 13.7 | 0.378 |  |
| FEV_1_/FVC (%) | 78.4 ± 4.9 | 79.4± 2.8 | 79.6 ± 5.0 | 78.6± 4.7 | 0.783 |  |
| Number of steps (/day) | 6087±2911 | 11898±4658* | 7151±1830 | 11599±5068* | 0.001 |  |
| Number of exercise bouts (/week) | 0.8±0.8 | 6.6±2.6* | 1.1±0.8 | 9.8±7.7* *** | <0.001 |  |

Note: ^†^Analyses performed on the natural logarithm.

Results were expressed as mean ± SD or median (interquartile range).

Values were analyzed by analysis of covariance with age as the covariate.

* *P* < 0.05 vs. the low-fitness/inactive group. ** *P* < 0.05 vs. the low-fitness/active group. *** *P* < 0.05 vs. the high-fitness/inactive group.

| **Table S3. CRP of the study participants during the study period.** | | | | | |
| --- | --- | --- | --- | --- | --- |
| **Characteristics** | **at baseline** | **aftter 3months** | **aftter 6 months** | **aftter 12 months** | ***P* value** |
| Low-fitness/Inactive Group (n = 19) | 0.10(0.04-0.23) | 0.08(0.05-0.15) | 0.09(0.05-0.21) | 0.12(0.07-0.23) | 0·602 |
| Low-fitness/Active Group(n=7) | 0.03(0.02-0.16) | 0.02(0.01-0.77) | 0.06(0.03-0.13) | 0.03(0.02-0.07) | 0.077 |
| High-fitness/Inactive Group(n=11) | 0.03(0.02-0.13) | 0.03(0.03-0.07) | 0.06(0.17-0.10) | 0.12(0.07-0.20) | 0.039 |
| High-fitness/Active Group (n = 16) | 0.04(0.02-0.08) | 0.07(0.03-0.18) | 0.05(0.02-0.11) | 0.05(0.02-0.80) | 0.238 |

Note: Results were expressed as median (interquartile range).

Analyses performed on the natural logarithm.

Values were analyzed by repeated measures ANOVA models were used to analyze.

| **Table S4. Characteristics of the study subjects at baseline and exercise volume of the study participants**  **during the study period.** | | | | | | |
| --- | --- | --- | --- | --- | --- | --- |
| **Characteristics** | **unimproved/inactive (n = 13)** | **unimproved/active (n = 8)** | **improved/inactive (n = 17)** | **improved/active (n = 15)** | ***P* value** |  |
| Age (years) | 50.2 ± 11.3 | 55.1 ± 9.6 | 53.2 ± 9.2 | 58.9 ± 6.7 | 0.104 |  |
| Duration of diabetes (years) | 5.7 ± 4.5 | 8.1 ± 7.7 | 6.5 ± 4.7 | 7.5 ± 5.8 | 0.913 |  |
| Smoking status (never/past/current) | 2/4/2 | 5/7/6 | 3/4/6 | 2/10/2 | 0.443 |  |
| Diabetes treatment |  |  |  |  |  |  |
| Sulphonylureas | 5 | 3 | 5 | 6 | 0.888 |  |
| α glucosidase inhibitors | 2 | 1 | 7 | 6 | 0.210 |  |
| biguanides | 2 | 1 | 4 | 0 | 0.295 |  |
| thiazolidines | 1 | 0 | 1 | 0 | 0.662 |  |
| glinides | 0 | 0 | 2 | 0 | 0.233 |  |
| Body mass index (kg/m^2^) | 24.7 ± 3.4 | 24.7 ± 3.6 | 26.9 ± 5.0 | 23.2 ± 3.5 | 0.154 |  |
| Total body fat (%) | 25.1 ± 5.6 | 24.8 ± 6.2 | 25.7 ± 9.0 | 20.4 ± 5.9 | 0.473 |  |
| Lean body mass (kg) | 51.3 ± 5.2 | 49.6 ± 5.3 | 53.8 ± 6.4 | 50.2 ± 7.1 | 0.392 |  |
| Systolic blood pressure (mmHg) | 134.2 ± 15.0 | 131.8 ± 15.0 | 143.0 ± 17.7 | 145.0 ± 23.2 | 0.302 |  |
| Diastolic blood pressure(mmHg) | 84.0 ± 8.3 | 82.0 ± 11.7 | 86.8 ± 10.5 | 85.2 ± 11.3 | 0.716 |  |
| Total cholesterol (mmol/l) | 5.66 ± 0.70 | 5.70 ± 1.02 | 5.02 ± 0.88 | 5.10 ± 0.90 | 0.149 |  |
| Triglyceride^a^ (mmol/l) | 1.89 (1.43–2.54) | 1.30 (1.00–1.90) | 1.77 (0.96–2.39) | 1.29 (0.76–1.85) | 0.421 |  |
| HDL cholesterol (mmol/l) | 1.27 ± 0.20 | 1.32 ± 0.32 | 1.21 ± 0.29 | 1.53 ± 0.32 ^‡^ | 0.048 |  |
| HbA1c (%) | 7.3 ± 1.4 | 7.3 ± 1.2 | 7.0 ± 1.2 | 7.0 ± 0.9 | 0.882 |  |
| Glycated albumin (%) | 23.2 ± 5.6 | 23.9 ± 4.8 | 20.6 ± 4.0 | 23.2 ± 3.6 | 0.240 |  |
| Peak O_2_ (ml/min/kg) | 27.2 ± 6.9 | 29.4± 3.8 | 24.4 ± 3.2* ^†^ | 25.8± 3.8* ^†^ | 0.057 |  |
| Peak O_2_ (%pred) (%) | 101.7 ± 24.7 | 114.4± 14.7 | 94.0± 12.8 | 102.9 ± 17.9 | 0.064 |  |
| FVC (%pred) (%) | 109.7 ± 16.8 | 101.0± 9.1 | 111.1± 10.7 | 111.4 ± 13.3 | 0.286 |  |
| FEV_1_ (%pred) (%) | 104.8 ± 15.2 | 101.8± 13.9 | 105.8 ± 11.9 | 111.0 ± 13.9 | 0.643 |  |
| FEV_1_/FVC (%) | 80.1 ± 5.4 | 80.1 ± 5.6 | 77.9 ± 4.4 | 78.2 ± 3.2 | 0.523 |  |
| Number of steps (/day) | 6060 ± 1409 | 11661 ± 6753* | 6796 ± 3222 ^†^ | 11706 ± 3748* ^‡^ | 0.001 |  |
| Number of exercise bouts (/week) | 1.1 ± 0.8 | 10.5 ± 10.2 * | 0.8 ± 0.8 ^†^ | 7.9 ± 3.8* ^‡^ | <0.001 |  |

Note: ^a^ Analyses performed on the natural logarithm.

Results were expressed as mean ± SD or median (interquartile range). Values were analyzed by analysis of

covariance with age as the covariate. * *P* < 0.05 vs. the unimproved /inactive group. ^†^ *P* < 0.05 vs. the

unimproved /active group. ^‡^ *P* < 0.05 vs. the improved/inactive group.
